# Water from Nitrodi’s Spring Induces Dermal Fibroblast and Keratinocyte Activation, Thus Promoting Wound Repair in the Skin: An In Vitro Study

**DOI:** 10.3390/ijms24065357

**Published:** 2023-03-10

**Authors:** Filomena Napolitano, Loredana Postiglione, Ilaria Mormile, Valentina Barrella, Amato de Paulis, Nunzia Montuori, Francesca Wanda Rossi

**Affiliations:** 1 Department of Translational Medical Sciences, University Federico II, 80131 Naples, Italy; filomena-napolitano88@hotmail.it (F.N.); lorposti@unina.it (L.P.); ilariamormile87@gmail.com (I.M.); depaulis@unina.it (A.d.P.); nmontuor@unina.it (N.M.); 2 Center for Basic and Clinical Immunology Research (CISI), WAO Center of Excellence, University Federico II, 80131 Naples, Italy; 3 CEINGE, Advanced Biotechnologies, 80145 Naples, Italy; valentinabarrella@hotmail.com

**Keywords:** antioxidant activity, dermal fibroblast proliferation, myofibroblasts, keratinocytes, Nitrodi’s water, skin aging, wound healing

## Abstract

The Romans knew of Nitrodi’s spring on the island of Ischia more than 2000 years ago. Although the health benefits attributed to Nitrodi’s water are numerous, the underlying mechanisms are still not understood. In this study, we aim to analyze the physicochemical properties and biological effects of Nitrodi’s water on human dermal fibroblasts to determine whether the water exerts in vitro effects that could be relevant to skin wound healing. The results obtained from the study indicate that Nitrodi’s water exerts strong promotional effects on dermal fibroblast viability and a significant stimulatory activity on cell migration. Nitrodi’s water induces alpha-SMA expression in dermal fibroblasts, thus promoting their transition to myofibroblast-protein ECM deposition. Furthermore, Nitrodi’s water reduces intracellular reactive oxygen species (ROS), which play an important role in human skin aging and dermal damage. Unsurprisingly, Nitrodi’s water has significant stimulatory effects on the cell proliferation of epidermal keratinocytes and inhibits the basal ROS production but enhances their response to the oxidative stress caused by external stimuli. Our results will contribute to the development of human clinical trials and further in vitro studies to identify inorganic and/or organic compounds responsible for pharmacological effects.

## 1. Introduction

The skin is the largest organ and functions as a protective barrier against environmental insults such as pathogens, chemicals, physical agents, and solar UVR. Moreover, the skin performs physiological functions such as immune defense, thermoregulation, sensing, endocrine functions, as well as metabolic effects [1,2,3]. The skin consists of three distinct layers: the epidermis, the dermis, and the hypodermis or subcutaneous tissue. The epidermis, the outermost level, is mainly made up of keratinocytes and contains melanocytes, Langerhans cells, and Merkel cells. The dermis, the internal layer that provides structural integrity, elasticity, and nutrition, is a connective tissue enriched with collagen and elastic fibers of a high fibroblast density; it also contains blood and lymphatic vessels, sebaceous glands, sweat glands, nerve endings, and hair follicles invaginated from the epidermis. The hypodermis or subcutaneous tissue consists of well-vascularized, loose, areolar connective tissue and adipose tissue functioning as a fat storage [4,5]. The integrity of the skin, constantly challenged by a wide variety of external factors, plays a pivotal role in maintaining physiological homeostasis and is of utmost importance for the viability of the inner tissues [6]. If left untreated, improper wound healing may lead to major disability or even death. Therefore, the proper, fast, and complete healing of wounds is of high priority for the viability of internal organs and thus for the survival of the organism.

It is important to underscore that the dermal fibroblasts represent the main regulators of skin homeostasis by interacting with the epidermis and other resident dermal cells, such as adipocytes, endothelial, neural, and inflammatory cells. Moreover, dermal fibroblasts are involved in various physio-pathological conditions, including wound healing, fibrosis, aging, and skin cancer [7,8].

Wound repair, a well-tuned biological process, occurs in three overlapping stages: inflammation, proliferation (including re-epithelialization, granulation tissue formation, and neovascularization), and remodeling [9]. Inflammation is the first stage of wound repair, which occurs immediately after tissue damage. The second stage—new tissue formation—is characterized by the activation of fibroblasts, some of which differentiate into myofibroblasts [10]. Myofibroblasts, characterized by the expression of alpha-smooth muscle actin (alpha-SMA), are contractile cells that, over time, start proliferating, migrate into the wound, and form the “granulation tissue”, which is rich in extracellular matrix (ECM) proteins. As fibroblasts begin to produce a new ECM, epithelialization is initiated. The epithelialization of wounds involves an orderly series of events in which keratinocytes migrate, proliferate, and differentiate to restore the epithelial barrier function [11]. Myofibroblasts support the growth of new blood vessels, reconstituting the wound bed and bringing the edges of a wound together [10]. Dysregulation in any phase of the wound healing cascade delays healing and may result in various skin pathologies, including non-healing or chronic ulceration [12,13].

The skin, as a critical protective barrier against the outside world, is also vulnerable to oxidative stress, potentially contributing to skin disorders ranging from functional impairments (skin cancer, dermatitis, and chronic and acute inflammatory diseases) to defects of aesthetic character, due to destruction of structural proteins and cellular changes, with the appearance of marks and lines of expressions and other signs typical of skin aging processes [14]. In particular, increased ROS levels determine DNA damage, inflammation, and the generation of matrix metalloproteinases (MMPs) that degrade collagen and elastin in the dermal skin layer [15,16]. Therefore, targeting oxidative stress may be an effective strategy for the anti-aging process and various skin disorders [17,18].

There is a growing body of evidence for the potential application of natural products (water, plants, and minerals), in addition to conventional treatment, to the enhancement of both acute and chronic wound healing [19,20,21,22].

We found it interesting that Nitrodi’s water affects the inflammatory microenvironment and enhances wound healing at the cellular level. The Nitrodi’s spring on the island of Ischia was known to Romans more than 2000 years ago as demonstrated by several marble votive reliefs found in this location, dated between the first century B.C. and the second century A.C. [23]. It has been found that in the spring, there was a school of medical hydrology attended by prominent physicians, such as Menippo, Aurelius Monnus, and Numerius Fabius [24]. Nitrodi’s water is classified as a mineral, hypothermal, sulfate alkaline and was extensively studied in the late 60s to evaluate the different modalities of exposition to the water. In particular, all studies showed that both internal and external use provided several benefits in the treatment of certain ailments [24], such as wound healing.

The beneficial effects of Nitrodi’s water can be observed particularly in the treatment of dermatitis and in almost all skin-related illnesses. Contact with this water is particularly beneficial for people suffering from venous ulcers because it acts on the nutritional processes of the tissues; hence, it is recommended for the treatment of lesions, fistulas, furuncles, burns, and ulcerative colitis. However, despite its long history, the biological mechanisms underlying the therapeutic effects on wound healing have not yet been elucidated, nor have clinical trials been performed to demonstrate its beneficial effects on patients with rheumatic or dermatologic disorders.

Recognizing the important role that traditional medicine continues to play, we have undertaken a preliminary in vitro study to clarify the biochemical and molecular mechanisms underlying the beneficial effects of Nitrodi’s spring water on skin inflammation and wound restoration.

## 2. Results

### 2.1. Composition of Nitrodi’s Water

Table 1 represents the main physical properties and mineral and organic composition of Nitrodi’s water.

The pH evaluation showed that Nitrodi’s water was slightly acidic, with a mean value of 6.33 on three measurements. Concerning the chemical composition of analyzed samples, the results indicated a medium mineral content, according to the Marotta and Sica classification [25,26]. The main components found were chloride (Cl^−^) with a mean value of 93 mg/L, sulfates (SO_4_^2−^) with a mean value of 204 mg/L, sodium (Na^+^) with a mean value of 174 mg/L, and calcium (Ca^2+^) with a mean value of 137 mg/L. These results obtained were comparable with those reported by Aversano, [27], which indicated an amount of 0.2200 g/L (220 mg/L), 0.1676 g/L (167.6 mg/L), and 0.1182 g/L (118.2 mg/L), for SO_4_^2−^, Na^+^, and Ca^2+^, respectively. However, the HCO_3_^−^ concentration obtained in this study (561 mg/L) was higher than that reported by Aversano (475.8 mg/L). Moreover, for bromide (Br^−^), the amount detected (0.18 mg/L) was in accordance with that reported by Inguaggiato (0.1–0.2 mg/L) [28]. On the other hand, the ammonium (NH_4_^+^) and total phosphorus (P) analyses showed values below LOQs, as well as for dissolved iron (Fe^2+^, Fe^3+^), and aluminum (Al^3+^). In addition to the parameters reported in Table 1, further verification analyses were carried out; the analyses included the evaluation of Polycyclic Aromatic Hydrocarbons (PAHs), Organochlorines (OCLs), Organophosphorus Pesticides (OPPs), Polychlorinated Biphenyls (PCBs), and Trihalomethanes (THMs), as determined by extraction and mass spectrometry analyses. The analyses showed for all analyte concentration values below LOQs. Therefore, according to the classification proposed by Nasermoaddeli and Kagamimori, based on its chemical composition and temperature, Nitrodi’s water was classified as mildly mineralized and hypothermal (20–30 °C) water [29]. In addition, as proposed by Marotta and Sica in the most widely accredited classification in Italy [25,26], as well as also by Mancioli [24], the results indicated that Nitrodi’s water falls into the category of medium-mineral waters of an essentially bicarbonate-sulfate-alkaline, alkaline-earthy, and hypothermal nature.

### 2.2. Nitrodi’s Water Sustains Dermal Fibroblast Viability via ERK Signaling Pathway

Dermal fibroblast proliferation plays a central role in the skin-repair process, inducing re-epithelialization through the replacement of disorganized collagen and elastin structures and the reposition of the extracellular matrix (ECM) in aged skin [30].

We assumed that the beneficial effects of Nitrodi’s water were, at least in part, due to the enhancement of dermal fibroblast activity. To test this hypothesis, we used BJ cells from the skin of normal foreskin as a cell model of the human skin.

**Figure 1:**
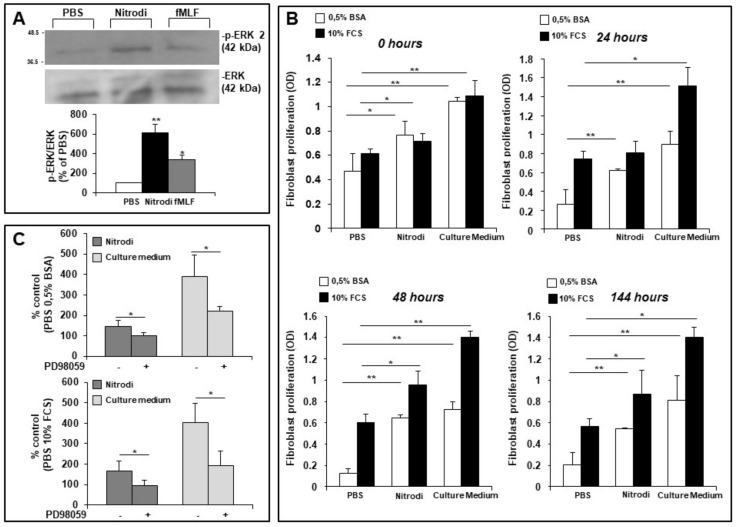
Effects of Nitrodi’s water on BJ cell proliferation. (**A**) Blot imaging and densitometric analysis. BJ cells, after incubation with PBS, Nitrodi, and fMLF for 5 min at 37 °C in a humidified (5% CO2) incubator, were lysed and subjected to Western blot analysis with anti phospho-ERK (p-ERK) antibody and then with the anti-ERK-2 antibody as a loading control. Histogram shows the levels of p-ERK 2 normalized to ERK value and expressed as a percentage of PBS control (**B**) Effects of PBS, Nitrodi, and culture medium on BJ cell proliferation. Cells were grown in 96-well plates for 0, 24, 48, and 144 h in the presence of 0.5% BSA (white column) or 10% FCS (black column). Cell viability was tested using the CellTiter 96 Aqueous One Solution Reagent. Cells treated with Nitrodi supplemented with 0.5% BSA or cells treated with culture medium supplemented with 0.5% BSA were compared to cells treated with PBS supplemented with 0.5% BSA at each time point. Cells treated with Nitrodi supplemented with 10% FCS and cells treated with culture medium supplemented with 10% FCS were compared to cells treated with PBS supplemented with 10% FCS at each time point. Error bars represent standard deviation of the mean of triplicate samples within one experiment. * *p* < 0.05; ** *p* < 0.001. (**C**) Effects of a specific inhibitor of the ERK pathway (PD98059) on BJ cell proliferation under starving and growing conditions. Cells were pre-treated with PD98059 and exposed to PBS (as control), Nitrodi’s water (dark grey columns), or culture medium (light grey columns) supplemented with 0.5% BSA or 10% FCS. Cell viability was tested using the CellTiter 96 Aqueous One Solution Reagent at 24 h after the treatments. Results are expressed as a percentage of the control (PBS 0.5% BSA or PBS 10% FCS). Values are mean ± SEM of three experiments. * *p* < 0.05.

As a preliminary step, we analyzed the effects of Nitrodi’s water by Western blot on the activation of extracellular signal-regulated protein kinases 1 and 2 (ERK 1/2), which are members of the mitogen-activated protein kinase super-family that regulates growth and cell-cycle progression. Serum-starved BJ cells were treated with PBS alone (as a negative control), Nitrodi, or fMLF (as a positive control) for ERK 1/2 activation [31,32,33]. Figure 1A shows that the incubation with Nitrodi for 5 min significantly increased the phosphorylation of ERK 2, but not of ERK 1, thus suggesting that Nitrodi’s water could act on BJ cell proliferation and cell-cycle progression.

Subsequently, we performed proliferation assays to analyze the effects of Nitrodi’s water on the rate of dermal fibroblast growth. BJ cells were incubated with Nitrodi supplemented with 0.5% BSA or 10% FCS, PBS supplemented with 0.5% BSA or 10% FCS, and culture medium supplemented with 0.5% BSA or 10% FCS, as negative and positive controls, respectively. The cell viability was measured at 0, 24, 48, and 144 h. These different time points have been used to simulate in vitro a hypothetical in vivo treatment of patients affected by skin failure syndrome. As shown in Figure 1B, Nitrodi’s water is able to sustain BJ cell survival, both in starving and growing conditions. This result was in agreement with ERK 2 induction by Nitrodi’s water, as observed in the previous experiment. An in-depth analysis of the proliferative course throughout the 144 h showed that culture medium-treated cells with 10% FCS produced an early peak at 24 h (O.D. 1.51 value) compared to T0 (O.D. 1.08 value), followed by a plateau of activity that persisted for 144 h. Nitrodi’s water-treated cells with 10% FCS exhibited a slight proliferative increase at 24 h (O.D. 0.81 value) and a peak at 48 h (O.D. 0.95 value) compared to T0 (O.D. 0.71 value). The mineral and ionic composition of Nitrodi’s water, supplemented with FCS, is able to deliver the ions, the nutrients, and the soluble factors essential for the maintenance of cell adherence and vitality. In fact, a decrease of cell count in PBS 10% FCS-treated cells was observed at the time points (at 48 h O.D. 0.601 value; at 144 h O.D. 0.56 value) subsequent to 24 h (O.D. 0.74 value) as expected as the lack of ions could induce cell detachment.

To better understand the role of ERKs in the cell proliferation, we analyzed the effects of PD98059, a potent and selective cell-permeable inhibitor of ERKs, on BJ proliferation. Serum-starved BJ cells were pre-incubated with PD98059 (50 µM) and then exposed to PBS, Nitrodi, and culture medium supplemented both 0.5% BSA and 10% FCS. The cell viability was measured after 24 h. As reported in Figure 1C, the cells treated with Nitrodi’s water and culture medium exhibited a significant reduction of proliferation in the presence of PD98059, both in the starving condition and in the growing condition. The inhibition of cell proliferation by PD98059 in culture medium-treated cells showed that these cells in physiologic conditions need ERK activation to proliferate. The same effects were observed when BJ cells, pre-treated with PD98059, were exposed to Nitrodi’s water. This result demonstrates that Nitrodi’s water does not alter or modify intracellular signaling pathways that regulate BJ proliferation.

All together, these preliminary data supported our hypothesis that Nitrodi’s water can offer several benefits for skin disorders by stimulating fibroblast survival via the MAPK/ERK signaling pathway. The decrease in cell count in the PBS negative control could be explained by the absence of ions, in particular, calcium and magnesium. This observation supports the hypothesis that the effects of Nitrodi’s water on fibroblast survival and viability are due to the mineral content, and further, future studies on the Nitrodi’s water microbiome will allow us to investigate its ability to stimulate skin cell proliferation.

### 2.3. Nitrodi’s Water Promoted In Vitro Wound Scratch Closure in Dermal Fibroblasts

Wound healing is a complex process modulated by an intricate signaling network involving several cell types, numerous growth factors, cytokines, and chemokines [34]. Successful healing depends on the optimal functioning of many diverse processes that lead to the generation of new tissue. The failure of any of these steps results in chronic inflammation. Fibroblasts play a key role in wound healing and tissue repair because the proliferation and subsequent migration of fibroblasts are of paramount importance for wound repair and healing [35].

**Figure 2:**
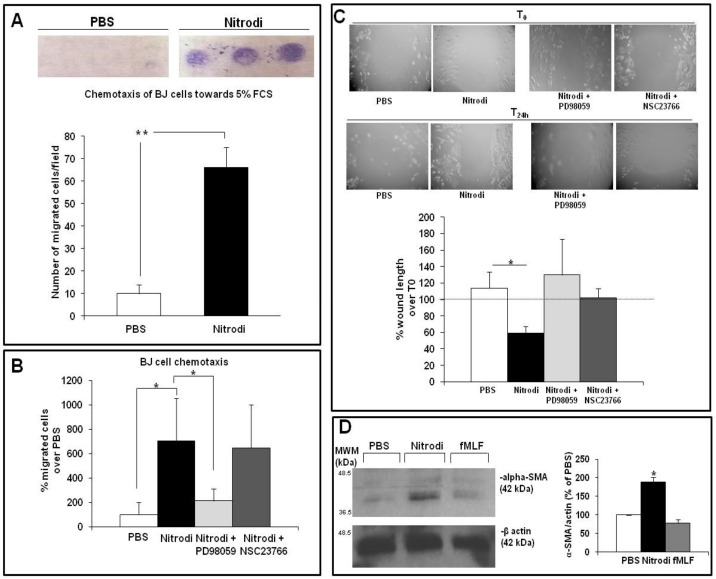
Effects of Nitrodi’s water on BJ cell migration. (**A**) Effects of PBS and Nitrodi on BJ cell chemotaxis. BJ cells were treated with PBS (white column) and Nitrodi (black column) and allowed to migrate towards 5% FCS for 24 h at 37 °C in a humidified (5% CO_2_) incubator. Error bars represent standard deviation of the mean of triplicate samples within one experiment. * *p* < 0.05; ** *p* < 0.001. The photograph of the membrane used for chemotaxis is shown at the top of the figure. (**B**) Effects of specific inhibitors of ERKs (PD98059) and Rac1 (NSC23766) on BJ cell chemotaxis. BJ cells were allowed to migrate towards 5% FCS for 24 h at 37 °C in a humidified (5% CO_2_) incubator. The data are expressed as a percentage of migrated cells over PBS (assumed as 100%). Error bars represent standard deviation of the mean of triplicate samples within one experiment. * *p* < 0.05. (**C**) Effects of PBS, Nitrodi, and Nitrodi in the presence of PD98059 and NSC23766 on BJ cell wound healing. Scratch images were acquired using inverted microscope and 4× magnification. Data were plotted and expressed as a percentage of the length of the wound over T0 (assumed as 100%). Error bars represent standard deviation of the mean of triplicate measurements within one experiment. * *p* < 0.05; ** *p* < 0.001 (**D**) Western blot and densitometric analyses of α-SMA expression in BJ cells. Relative α-SMA expressions were normalized to the respective value for total β-actin. Histogram shows the levels of α-SMA normalized to β-actin values and expressed as a percentage of PBS control.

To analyze the effects of Nitrodi’s water on in vitro wound-scratch closure, we first tested its capability to induce directional migration (i.e., cell motility in one direction) after 24 h of treatment through a chemotaxis assay in BJ cells. The study of fibroblast migration was performed in a Boyden modified chamber using 5% FCS as a generic chemoattractant. In these assays, the membrane coating with fibronectin also allowed us to investigate the capacity of fibroblasts to enter the wound matrix in response to Nitrodi’s water. Figure 2A shows that BJ cells treated with Nitrodi were readily able to migrate towards 5% FCS and to invade through fibronectin-coated membrane as compared to cells treated with PBS alone.

To clarify which signaling pathways are involved in directional fibroblast migration promoted by Nitrodi’s water, we further investigated the respective roles of ERKs and Rac1 as the main signaling mediators involved in cell migration. To this end, the pre-treatment of BJ cells with the specific inhibitors of ERKs (PD98059) and Rac1 (NSC23766) was carried out for 2 h at 37 °C. Thus, chemotaxis assay was performed. As shown in Figure 2B, when the cells were pre-treated with PD98059 (50 µM) and then exposed to Nitrodi’s water, the migration ability was obviously impaired. Conversely, the pretreatment with NSC23766 (25 µM), a widely used inhibitor of Rac1 activation, did not exert any effects on Nitrodi’s water-accelerated fibroblast migration. These data indicated that ERK pathway plays a crucial role in fibroblast chemotaxis promoted by Nitrodi’s water, as already demonstrated in the previous analysis of fibroblast proliferation.

After the investigation of fibroblast unidirectional migration through chemotaxis assay, we examined the effects of Nitrodi’s water on wound-healing scratch assay, as a model of non-oriented cell migration. After scratching, BJ cells were incubated for 24 h with PBS, Nitrodi, Nitrodi’s water containing PD98059 (50 µM), and Nitrodi’s water containing NSC23766 (25 µM). Figure 2C shows the images acquired under the microscope at 4x magnification over time and the graph containing a data analysis of the images. In the graph, BJ cell migration was expressed as the percent of wound length over T0 (assumed as 100%). Nitrodi’s water alone enhanced BJ cell migration at 24 h as compared to cells treated with PBS, whereas the presence of ERK and Rac1 inhibitors impaired the migration/invasion of BJ cells into the wound. These results indicate that Nitrodi’s water promotes cell motility through both ERKs and Rac1 in the non-oriented migration assay, whereas ERK activation is preferentially required for unidirectional migration (chemotaxis assay) towards chemoattractant [36,37].

Finally, Nitrodi’s water not only exerts a strong chemotactic effect on BJ cells through the promotion of unidirectional migration but also induces a significant improvement in wound scratch closure.

### 2.4. Nitrodi’s Water Promoted Dermal Fibroblast Differentiation through Alpha-SMA Induction

Tissue damage activates fibroblasts and differentiates them into myofibroblasts. These cells can contract and actively produce ECM proteins to enable wound closure [38]. Alpha smooth-muscle actin (alpha-SMA) expression is a marker of myofibroblast differentiation [39].

Thus, we investigated the impact of the Nitrodi’s water treatment on BJ cell transition to myofibroblasts. To this end, we used a Western blot analysis to evaluate the expression levels of alpha-SMA in BJ cells after incubation with PBS as a vehicle control and Nitrodi and fMLF (10^−4^ M) as a positive control for alpha-SMA induction [31,40].

As shown in Figure 2D, the alpha-SMA expression level increased significantly in BJ cells in response to Nitrodi compared to PBS-treated controls.

Our data indicated that Nitrodi’s water induced the differentiation of BJ cells into myofibroblasts, which are necessary to optimize skin repair.

### 2.5. Nitrodi’s Water Promotes ECM Protein Deposition in Dermal Fibroblasts

Upon injury, dermal fibroblasts migrate into wound granulation tissue and differentiate into myofibroblasts, which play a central role in the wound contraction and deposition of ECM proteins. To investigate the possibility that the synthesis of ECM could be promoted by Nitrodi’s water, we evaluated the deposition of vitronectin, fibronectin, and collagen type I by in situ ELISA in basal conditions (PBS-treated cells) and after treatment with Nitrodi’s water in BJ cells.

**Figure 3:**
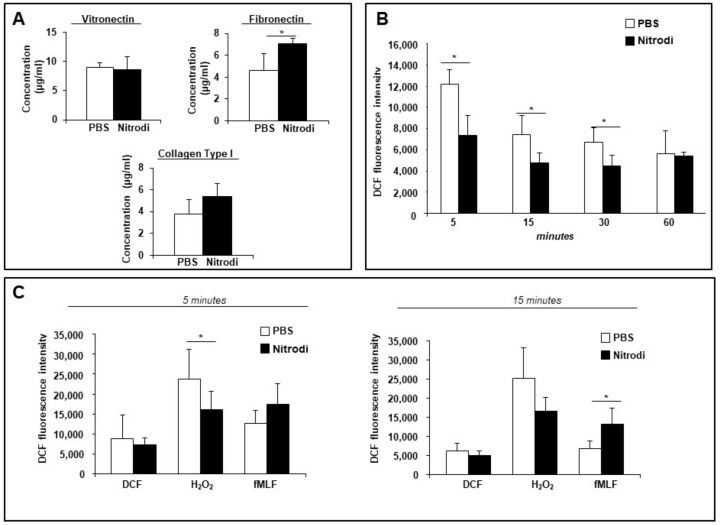
Effects of Nitrodi’s water on ECM deposition and ROS production by BJ cells. (**A**) Analysis of effects of Nitrodi’s water on BJ cell ECM deposition. ECM proteins vitronectin, fibronectin, and collagen type I were quantified by in situ ELISA. Protein concentration measurements were performed using calibration curves generated by plates coated with purified ECM components at different concentrations. Error bars represent standard deviation of the mean of triplicate samples within one experiment. * *p* < 0.05. (**B**) Cells were plated in a 96-well plate and treated with DCFH-DA (2′, 7′-dichlorodihydrofluorescein diacetate). At the end of incubation, cells were treated with PBS alone (white columns) or Nitrodi (black columns). ROS release was measured as dichlorofluorescein (DCF) fluorescence intensity at 5, 15, 30, and 60 min. Results are expressed as the mean fluorescence intensity of DCFH-DA-loaded cells less the values of the mean fluorescence intensity of DCFH-DA-unloaded cells. Error bars represent standard deviation of the mean of triplicate samples within one experiment. * *p* < 0.05 (**C**) Cells were plated in a 96-well plate and incubated with DCFH-DA. At the end of incubation, cells were treated with medium alone (white columns), H_2_O_2_ (black columns), or fMLF (grey columns) in presence of PBS or Nitrodi. ROS release was measured as dichlorofluorescein (DCF) fluorescence intensity at 5 and 15 min. Results are expressed as the mean fluorescence intensity of DCFH-DA-loaded cells less the values of the mean fluorescence intensity of DCFH-DA-unloaded cells. Error bars represent standard deviation of the mean of triplicate samples within one experiment. * *p* < 0.05.

As shown in Figure 3A, BJ cells exposed to Nitrodi’s water exhibited a significant increase in fibronectin deposition (7.06 ± 0.49 μg/mL) compared to the control (4.6 ± 1.58 μg/mL). No differences between the control (9.03 ± 0.80 μg/mL) and cells treated with Nitrodi’s water (8.6 ± 2.23 μg/mL) were detected in vitronectin deposition. A slightly increasing trend in cells treated with Nitrodi’s water (5.4 ± 1.15 μg/mL) was recorded in collagen deposition compared to control (3.7 ± 1.35 μg/mL).

These data showed that Nitrodi’s water promoted the expression of fibronectin and, to a lesser extent, collagen, which are major proteins of ECM and are critical for both structural support and cell adhesion [41]. Instead, Nitrodi’s water did not exert any effect on the deposition of vitronectin, which is a provisional matrix component, mostly increased in patho-physiologic settings associated with acute inflammation [42].

Taken together, these results indicate that Nitrodi’s water contributes to the production of the main ECM components, particularly fibronectin, which plays a crucial role in ECM formation and in re-epithelialization during wound healing.

### 2.6. Nitrodi’s Water Exhibited Anti-Oxidant Properties in Dermal Fibroblasts

Reactive oxygen species (ROS) play a central role in both chronological aging and photo-aging. Oxidative stress promotes tissue inflammation through the upregulation of genes that encode pro-inflammatory cytokines and the sustained activation of the NF-ΚB pathway. This low-grade chronic inflammation and the up-regulation of pro-inflammatory mediators are referred to as skin inflamm-aging [43,44,45,46].

Several natural compounds have been studied in order to evaluate their effects on skin inflamm-aging, but further investigations are needed. Here, we examined the effects of Nitrodi’s water on the ROS release from the BJ cell line, both in the absence and presence of oxidant stimuli. To this aim, BJ cells were loaded for 30 min with dichloro-dihydro-fluorescein diacetate (DCFH-DA), the most widely used probe for detecting intracellular oxidative stress, and then treated with PBS (as a negative control) or with Nitrodi for 5, 15, 30, and 60 min. Figure 3B shows that, at all different time points, BJ cells treated with Nitrodi’ water had significantly lower intracellular ROS levels compared to BJ cells treated with PBS, indicating that Nitrodi’s water can be considered as a promising source of natural antioxidants and is useful for the treatment of chronic inflammation and anti-aging strategies.

Exogenous H_2_O_2_ is known to induce oxidative stress and cell death by oxidizing lipids and proteins [47]. Moreover, different cell types, such as fibroblasts, generate small amounts of ROS via the activation of NADPH oxidase in response to growth factors, cytokines, and G-protein-coupled receptor (GPCRs) agonists. Of importance, these endogenous ROS serve as second messengers to activate multiple intracellular signaling pathways essential to cell physiological responses, including the growth, migration, and modification of the ECM [48]. We have previously demonstrated that fMLF, a bacterial analog peptide that binds GPCRs, is able to induce ROS generation by NADPH oxidase complex activation, playing an important role in antimicrobial host defense and inflammation [31,40]. Thus, we performed an ROS detection assay in the presence of two different stimuli, H_2_O_2_, as an exogenous oxidative stress, and fMLF.

As reported in Figure 3C, in H_2_O_2_-loaded BJ cells, Nitrodi’s water treatment exhibited a protective role against oxidative stress as compared to H_2_O_2_-loaded BJ cells in PBS solution as ROS levels were lower in Nitrodi-treated cells than in PBS-treated cells. Therefore, Nitrodi’s water could contribute to improving the oxidative response under stress conditions, thus, avoiding an uncontrolled ROS generation.

Conversely, the presence of Nitrodi’s water ensured the ROS production in response to fMLF, demonstrating that Nitrodi’s water did not affect the maintenance of redox homeostasis in response to various cellular signaling pathways.

### 2.7. Nitrodi’s Water Promotes Multi-Oriented, but Not Directional, Epidermal Keratinocyte Migration

Epidermal keratinocytes, the predominant cell type in the skin epidermis, are among the front line of skin defense. Keratinocytes contribute to ensuring efficient and harmonious wound healing through coordinated action with fibroblasts and immune cells. In particular, keratinocytes are the executors of the re-epithelialization phase, whereby keratinocytes migrate, proliferate, and differentiate to restore the epidermal tissue [49].

**Figure 4:**
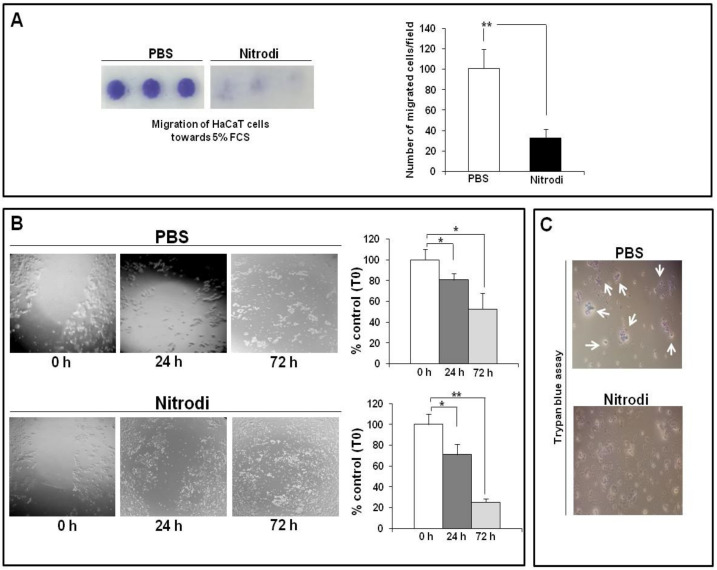
Effects of Nitrodi’s water on HaCaT cell migration. (**A**) Effects of PBS and Nitrodi on HaCaT cell chemotaxis. Cells treated with PBS (white column) and Nitrodi (black column) were allowed to migrate in response to 5% FCS for 24 h at 37 °C in a humidified (5% CO_2_) incubator. Error bars represent standard deviation of the mean of triplicate samples within one experiment. ** *p* < 0.001. (**B**) Effects of Nitrodi’s water on HaCaT cell wound healing. Scratch images were acquired using inverted microscope and 4× magnification. Data were plotted and expressed as a percentage of the length of wound size over T0 (assumed as 100%). Error bars represent standard deviation of the mean of triplicate measurements within one experiment. * *p* < 0.05; ** *p* < 0.001 (**C**) In situ Trypan blue staining of HaCaT cells. Photographs show cells treated for 72 h with PBS and Nitrodi’s water. The images were acquired using inverted microscope and 10× magnification. The arrows indicate the unviable cells.

The initial phase of re-epithelialization results in keratinocyte migration from a surrounding tissue. Hence, we tested the capability of Nitrodi’s water to induce directional migration of HaCaT cells, as a model of human epidermal keratinocytes. Figure 4A shows that HaCaT cells treated with Nitrodi’s water exhibited a less vigorous migratory phenotype, as compared to control. Contrary to data obtained in BJ cells, it would seem that Nitrodi’s water exerts an inhibitory effect on HaCaT cell migration.

Surprised by these results, we performed a wound-healing assay to analyze the effects of Nitrodi’s water on non-oriented migration of HaCaT cells. After scratching, HaCaT cells were incubated with PBS or Nitrodi for 24 and 72 h. Figure 4B shows the images acquired at different time points and related plots for wound-size quantification. Cell migration rate was expressed as a percentage of the length of wound size over T0 (assumed as 100%). Both cells treated with PBS and Nitrodi’s water exhibited the ability to invade the wound site, but our experiments illustrated how the two conditions, PBS/distilled water and PBS/Nitrodi’s water (equal in the solute but different in the solvent), influenced cell morphology and density differently.

In light of these findings, we evaluated the effects of Nitrodi’s water and PBS on cell viability at 72 h through in situ Trypan blue staining. In Trypan blue assay, dead cells are stained because the dye cannot permeate the intact cell membrane. As shown in Figure 4C, Nitrodi’s water did not exhibit any cytotoxic effects on keratinocyte monolayer subjected to scratching, whereas the cells in PBS exhibited an intense trypan blue staining.

Importantly, the results of these experiments showed that Nitrodi’s water did not alter the ability of epithelial cells to maintain close contact and continuity with each other and move forward in a collective way to restore the epithelial barrier at the wound site [50].

### 2.8. Nitrodi’s Water Supports Epidermal Keratinocyte Viability and Survival

Wound repair requires keratinocyte proliferation to restore the epithelial barrier [51]. Growth factors produced as a result of injury are released by several cell types to stimulate keratinocyte proliferation, and integrins on the keratinocyte surface enhance the accumulation of intracellular signaling mediators in order to enhance proliferation [52].

**Figure 5:**
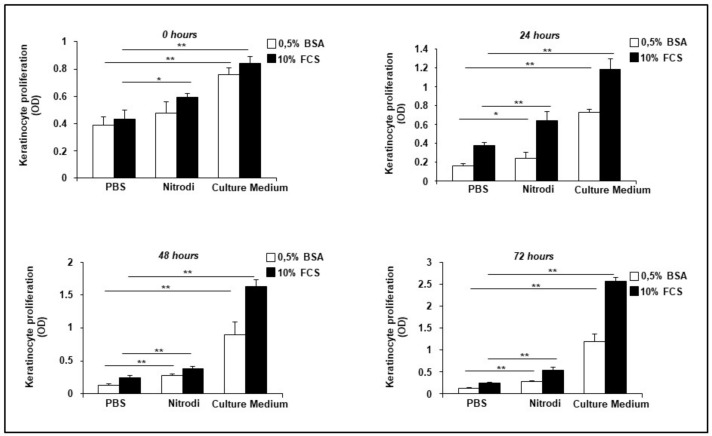
Effects of Nitrodi’s water on HaCaT cell proliferation. Cells were grown in 96-well plates for 0, 24, 48, and 72 h, in the presence 0.5% BSA (white column) and 10% FCS (black column). Cell viability was tested by CellTiter 96 Aqueous One Solution Reagent. Cells treated with Nitrodi supplemented with 0.5% BSA and cells treated with culture medium supplemented with 0.5% BSA were compared to cells treated with PBS supplemented with 0.5% BSA at each time point. Cells treated with Nitrodi supplemented with 10% FCS and cells treated with culture medium supplemented with 10% FCS were compared to cells treated with PBS supplemented with 10% FCS at each time point. Error bars represent standard deviation of the mean of triplicate samples within one experiment. * *p* < 0.05; ** *p* < 0.001.

To evaluate whether Nitrodi’s water exerts any effect on keratinocyte proliferation, we performed in vitro analyses of cell proliferation in the HaCaT cell line. HaCaT cells were incubated with Nitrodi supplemented with 0.5% BSA and 10% FCS, or with PBS supplemented with 0.5% BSA and 10% FCS, and culture medium supplemented with 0.5% BSA and 10% FCS to reproduce the starving and growing condition, respectively. As shown in Figure 5, Nitrodi’s water had significant stimulatory effects on keratinocytes, as HaCaT cells treated with Nitrodi 0.5% BSA showed a significant increase in cell proliferation as compared to BJ cells treated with PBS 0.5% BSA. This significant proliferation increase was confirmed by a comparison of the treatment with Nitrodi 10% FCS and HaCaT cells treated with PBS 10% FCS. The increase in cell proliferation induced by Nitrodi’s water was observed when Nitrodi-treated cells were compared to the negative control (PBS), but not to the positive control (culture medium). It is important to underline that in the culture medium, there are vitamins, nutrients, and glucose that cells need to survive and proliferate, unlike in Nitrodi’s water. Despite this, during the 72 h curve, no significant decrease in cell number was observed in Nitrodi-treated cells as opposed to PBS. Therefore, a decrease in cell number was observed during the 72 h curve in both PBS 0.5% BSA (0.38 O.D. vs. 0.13 O.D.) and PBS 10% FCS (0.43 O.D. vs. 0.24 O.D.). On the contrary, cell survival was slightly affected in Nitrodi 0.5% BSA (0.47 O.D. vs. 0.27 O.D.) and was not affected in Nitrodi 10% FCS (0.59 O.D. vs. 0.53 O.D.). The conclusion would be that Nitrodi’s water supports cell survival in keratinocytes, and the beneficial effects are probably due to mineral content and soluble factors that are still unidentified.

### 2.9. Nitrodi’s Water Elicits an Acute Stress Response to Pro-Inflammatory Agents in Keratinocytes

Cutaneous perturbations created by acute exposome factors (environmental and/or internal) induce responses to protect the organism and re-establish homeostasis [53]. Epidermal keratinocytes, which occupy the outermost layer of the skin, are always exposed to external stimuli, which constantly generate ROS in the cells [54].

**Figure 6:**
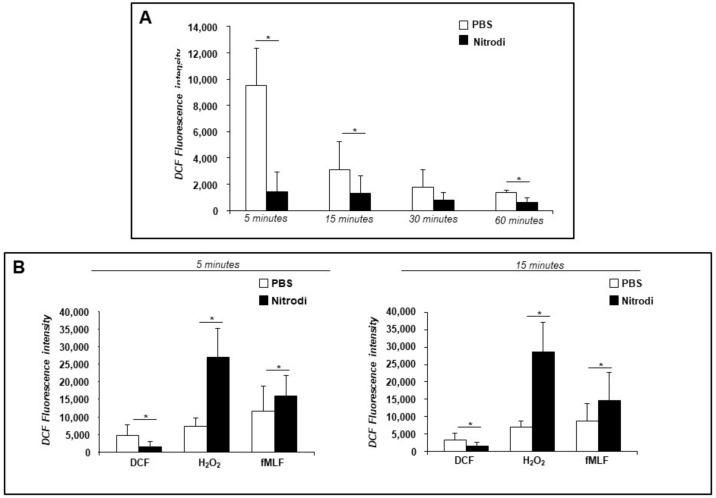
Effects of Nitrodi’s water on ROS production by HaCaT cells. (**A**) Cells were plated in a 96-well plate and treated with DCFH-DA (2′, 7′-dichlorodihydrofluorescein diacetate). At the end of incubation, cells were treated with PBS alone (white columns) or Nitrodi (black columns). ROS release was measured as dichlorofluorescein (DCF) fluorescence intensity at 5, 15, 30, and 60 min. Results are expressed as the mean fluorescence intensity of DCFH-DA-loaded cells less the value of the mean fluorescence intensity of DCFH-DA-unloaded cells. Values are the mean ± SEM of three experiments performed in triplicate. * *p* < 0.05. (**B**) Cells were plated in a 96-well plate and incubated with DCFH-DA. At the end of incubation, cells were treated with medium alone (white columns), H_2_O_2_ (black columns) or fMLF (grey columns) in presence of PBS or Nitrodi. ROS release was measured as dichlorofluorescein (DCF) fluorescence intensity at 5 and 15 min. Results are expressed as the mean fluorescence intensity of DCFH-DA-loaded cells less the value of the mean fluorescence intensity of DCFH-DA-unloaded cells. Values are the mean ± SEM of three experiments performed in triplicate. * *p* < 0.05.

Then, we analyzed the effects of Nitrodi’s water on oxidant activity in HaCaT cells. HaCaT cells were loaded for 30 min with DCFH-DA, and then treated with PBS (as a negative control) or with Nitrodi for 5, 15, 30, and 60 min. As shown in Figure 6A, in the absence of external stimuli, the measurement of intracellular ROS in Nitrodi-treated HaCaT cells revealed significantly decreased ROS levels compared to untreated cells, as already observed in BJ cells.

Subsequently, we performed an ROS detection assay in the presence of H_2_O_2_ to cause oxidative damage stress, and we used a high concentration of fMLF (10^−4^ M) as a typical pro-inflammatory agent. HaCaT cells in PBS solution did not respond or responded poorly to H_2_O_2_ and fMLF, both at 5 and 15 min (Figure 6B). Instead, HaCaT cells treated with Nitrodi’s water early generated a significant amount of ROS in response to both H_2_O_2_ and fMLF, unlike what was observed in dermal fibroblasts (paragraph 2.6.).

These data showed that Nitrodi’s water enhances the antioxidant defense-system potential under steady-state conditions, whereas it confers pro-oxidant phenotype in response to pro-inflammatory stimuli.

## 3. Discussion

Bath therapy is an effective complementary approach to the management of several low-grade inflammation- and stress-related pathologies, particularly rheumatic and metabolic diseases [29]. However, despite the notable clinical benefits of these therapies, their role in modern medicine is still controversial, especially since the biological mechanisms underlying these benefits have not yet been completely clarified.

Due to interesting peculiarities of water from Nitrodi’s spring, their clinical application is of great interest. Since Roman times, the therapeutic effectiveness of Nitrodi’s water has been known in various skin diseases, such as psoriasis, contact dermatitis, acne, and eczema [24].

This preliminary study aims to analyze the physicochemical composition and the biological effects of Nitrodi’s water at cellular level. To this end, Nitrodi’s water was taken from the historical thermal resort of Ischia, Italy. Firstly, the physicochemical analysis showed that Nitrodi’s water is a medium-mineral water, according to the Marotta and Sica [35,36]. The constituent mineral elements, such as silica, sulphates, sodium, and calcium, might be responsible for pharmacological actions. It is well-established that calcium plays a central role in wound healing and keratinocyte differentiation in the skin [55]. Nitrodi’s water contains conspicuous levels of calcium (137 mg/L), which could be responsible for benefits in the management of some skin diseases such as rosacea and psoriasis. Nitrodi’s water also contains silica (82 mg/L), which is involved in skin wound repair and plays a protective role on the connective tissue and cartilage. Silica might be also responsible for the suppression of pro-inflammatory cytokines [56]. Bicarbonate helps neutralize acid valences in cases of excessive muscle work; therefore, bicarbonate concentration [561 mg/L] in Nitrodi’s water could explain the clinical efficacy in the rehabilitation of the musculoskeletal. Moreover, the ability of Nitrodi’s water to improve skin regeneration could be due to calcium, magnesium, and bicarbonate, as previously established [57]. Sulfur exerts well-known anti-inflammatory and anti-oxidative activities in HaCaT cells [58]; therefore, the anti-inflammatory effect of Nitrodi’s water might be attributed to the sulfur content. In addition to an analysis of minerals dissolved in Nitrodi’s water, the evaluation of the organic fraction was performed, showing values below LOQs for PAHs, OCLs, OPPs, PCBs, and THMs. These data allowed us to assess the absence of toxicity risks since the absorption of these compounds into the skin, into the oral mucosa, or by inhalation is considerable [59].

The biochemical and molecular characteristics of the water from Nitrodi’s spring and its effects on skin inflammation and wound restoring were evaluated by using the BJ cell line as a model of human dermal fibroblasts. Moreover, a series of experiments were performed in HaCaT cells, as a model of skin keratinocytes, in order to have a complete picture of the effects of Nitrodi’s water in wound repair. In the research methods of this study, a PBS solution is used as a negative control. PBS reproduces the pH, the osmolarity, and the ion concentration of human body. Unlike water, PBS avoids cells rupturing due to osmosis, even though it was formulated without the addition of vitamins and amino acids. The absence of ions in PBS, such as calcium and magnesium, allowed us to investigate the role of Nitrodi’s water mineral content at cellular level. However, the beneficial effects of Nitrodi’s water on the skin are not only attributable to the mineral content, but it is conceivable that soluble factors or active compounds contained in these waters, in association with minerals, are effectively responsible for the multiple therapeutic effects. Therefore, the effects on fibroblasts and keratinocytes only partially reproduce the therapeutic potential of Nitrodi’s water.

Skin wound healing is a complex process that can be divided into at least three continuous and overlapping processes: an inflammatory reaction, a proliferative process leading to tissue restoration, and tissue remodeling [9]. In the current study, we demonstrate that the water from Nitrodi’s spring supports BJ cell survival, and this activity is mediated by ERK signaling pathway.

To investigate whether Nitrodi’s water exerts any effect on fibroblast migration, two different types of tests were performed, chemotaxis and wound healing. A chemotaxis assay was exploited to analyze the unidirectional movement of fibroblasts treated with Nitrodi’s water; whereas a wound healing assay was performed to study the ability of Nitrodi’s water to induce multi-oriented cell migration. Nitrodi’s water promotes both uni- and multi-directional migration in BJ cells. The ERK signaling pathway is required for chemotaxis, and both ERK signaling and Rac1 activation are required for fibroblast wound healing. Likely, ERK signaling moves fibroblasts between points, whereas Rac1 allows cells to explore their local environment and thus migrate into the wound. ERK signaling seems to coordinate the activity of Rac1 in fibroblast non-directional migration, as demonstrated by the inability of Nitrodi’s water to close the wound in the presence of both specific inhibitors of ERK and Rac1.

Most importantly, Nitrodi’s water induced the expression of the alpha-SMA protein, thus stimulating the transition to myofibroblasts that are necessary for wound contraction. Therefore, when the fibroblasts migrate into the wound site from the surrounding tissue, they become activated, transform into myofibroblasts, and begin synthesizing extracellular matrix (ECM) proteins, mainly collagen. ECM deposition is essential for proper wound healing; in BJ cells, Nitrodi’s water is able to induce the synthesis of fibronectin, an adhesive molecule that plays a crucial role in wound healing, particularly in ECM formation and tissue regeneration.

At baseline conditions, in vitro treatment of BJ cells with Nitrodi’s water reduced ROS production, suggesting that it keeps ROS levels low in the absence of stimuli, as compared to PBS. Moreover, the Nitrodi’s water treatment exhibited a protective role against oxidative stress caused by H_2_O_2_ in BJ cells, whereas in fMLF-stimulated cells it was able to preserve NADPH enzymatic complex activation in response to inflammatory stimuli.

The tissue-repair process not only involves the generation of connective tissue via fibroblasts and the formation of new vessels via endothelial cells but also re-epithelialization via keratinocytes. Epithelialization is used as a defining parameter of a successful wound closure [60]. Our data show that Nitrodi’s water exerts paradoxical effects on keratinocyte migration. In chemotaxis towards a chemical signal, Nitrodi’s water inhibits cell migration; whereas, in the wound healing assay, the cells treated with Nitrodi’s water have the ability to respond effectively to mechanical stimuli generated by scraping off an area covered by cells. There may be several explanations for this phenomenon. It is conceivable that Nitrodi’s water strengthens the function of the physical, but not chemical, barrier of skin keratinocytes at the interface between the body and the environment; or it could happen that Nitrodi’s water contributes to wound closure by inducing keratinocyte proliferation rather than cell migration. In fact, further experiments show that Nitrodi’s water exerts significant stimulatory effects on cell survival in epidermal keratinocytes. Moreover, Nitrodi’s water inhibited the basal ROS production of epidermal keratinocytes, as already observed in dermal fibroblasts, but enhanced their response to the oxidative stress caused by external stimuli.

Finally, the protective effects of Nitrodi’s water have not been previously described and may explain some of the positive effects of Nitrodi’s water in wound treatment. Further studies need to be conducted in order to identify which components of Nitrodi’s water aid in the wound healing process and ROS neutralization.

Although this study demonstrated potential mechanisms of Nitrodi’s water in promoting wound healing, in vitro wound healing assays cannot mimic the complexity of the conditions that take place in vivo. Therefore, data obtained from in vitro assays should not be considered definitive and should be corroborated through in vivo models in order to exclude that Nitrodi’s water could induce fibroproliferative disorders, such as keloids and/or hypertrophic scarring. An investigation of which mineral component(s) in Nitrodi’s water are required for a therapeutic effect should be investigated in the future. Furthermore, the overall non-pathogenic bacteria populations of Nitrodi’s water, termed microbiota, may be responsible for its regenerative properties. These properties may be related to the production of so-far-unknown substances that promote regeneration, probably in synergy with macro- and micro-mineral elements of the spring water [61]. Importantly, the applied concentrations to experimental solutions do not match the concentration found in waters taken from the natural spring. Besides, the filtration of Nitrodi’s water alters the real composition of these waters, but sterile conditions have been required to perform this preliminary in vitro study. When searching for evidence in thermal medicine, the best strategy is to conduct human clinical trials, but in vitro evaluations of thermal waters are insufficient.

In conclusion, the elucidation of the biological mechanisms underlying the benefits of Nitrodi’s water will contribute to the development of potential therapies for skin diseases. Nitrodi’s water could be a promising anti-inflammatory agent for the skin, as well as a potential wound-healing therapeutic agent. In addition, the antioxidant properties of Nitrodi’s water could be exploited to prevent symptoms related to photo-induced aging of the skin.

## 4. Materials and Methods

### 4.1. Peptides and Chemicals

*N*-Formyl-methionyl-leucyl-phenylalanine (fMLF), PD98059 (1,4-diamino-2,3-dicyano-1,4-bis[phenylthio] butadiene), and NSC23766 were purchased from Calbiochem (La Jolla, CA, USA). Protein concentration was determined with a modified Bradford assay (Bio-Rad Laboratories). ECL Plus was obtained from GE Healthcare (Buckinghamshire, UK), and 29, 79-dichlorodihydrofluorescein diacetate (DCFH-DA) was obtained from Molecular Probes (Invitrogen, Paisley, UK). The protease and phosphatase inhibitors cocktails were obtained from Calbiochem (La Jolla, CA, USA). Mouse anti-phospho-ERK (catalog number sc-7383), rabbit anti-ERK 2 (catalog number sc-154), mouse anti-collagen type I (catalog number sc-166865), and mouse anti-fibronectin (catalog number sc-271098) were obtained from Santa Cruz Biotechnology (Santa Cruz, CA, USA); mouse anti-alpha-SMA (catalog number A 2547) was from Sigma-Aldrich (St. Louis, MO, USA); mouse anti-vitronectin (catalog number MAB1945) was from Chemicon International (Temecula, CA, USA); rabbit anti-actin (catalog number A2066) was from Sigma-Aldrich (St. Louis, MO, USA). Secondary anti-mouse and anti-rabbit Abs coupled to HRP were from Bio-Rad (Munchen, Germany). For the chemotaxis assay, 8-μm-pore polycarbonate membranes (Nucleopore, Pleasanton, CA, USA) coated with 10 μg/mL of fibronectin (Roche, Mannheim, Germany) were used. CellTiter 96 Aqueous One Solution Reagent was from Calbiochem (San Diego, CA, USA). Vitronectin was purchased from BD Biosciences (Bedford, MA, USA); collagen type I was purchased from Chemicon International (Temecula, CA, USA). Trypan blue solution was from Sigma-Aldrich (St. Louis, MO, USA).

### 4.2. Analysis of Nitrodi’s Water Composition

The physicochemical characteristics were carried out using standard analytical methods (analyses were performed in accordance with Standard Methods for Examination of Water and Wastewater prepared and published jointly by APHA (American Public Health Association), AWWA (American Water Works Association), and AEF (American Environment Federation). The pH was evaluated using a Mettler Toledo-SevenExcellence pH/Cond meter S470-Std-K. Bicarbonate (HCO_3_^−^) and iodide (I^−^) were determined by the titrimetry method. Ammonium (NH_4_^+^), total phosphorus (*p*), and silica (SiO_2_) were evaluated by UV-Vis spectroscopy. For a determination of chlorides (Cl^−^) and sulfates (SO_4_^2−^), a DIONEX Integrion HPIC^TM^ ICS1100 ion chromatography system (Thermofisher) was used [62]. The Dionex 1100 was equipped with a Dionex EGC 500 KOH RFIC^TM^, a potassium hydroxide (KOH) eluent generator cartridge, and an IonPac AS27 RFIC^TM^ (4 × 250 mm) (Thermo Fisher Scientific, Waltham, MA, USA) analytical column. Deionized water (>18 MΩ) was used for generating the eluent. For the analysees of sodium (Na^+^), potassium (K^+^), calcium (Ca^2+^), magnesium (Mg^2+^), dissolved iron (Fe^2+^, Fe^3+^), stronzium (Sr^2+^), litium (Li^+^), and alluminium (Al^3+^), water samples were filtered using GF/F glass fiber filters (47 mm × 0.7 µm; Whatman, Maidstone, UK) and acidified with 1% HNO_3_/HCl [63,64]. A Thermo Scientific^TM^ ICAP^TM^ RQ inductively-coupled plasma mass spectrometer (Q-ICP-MS), operating by Qtegra^TM^ Software (Version 2.7.2425.65), was used. The operating conditions of the Q-ICP-MS equipment were optimized using a tuning solution (Ba, Bi, Ce, Co, In, Li, U 1.00 µg/L, Thermo Scientific Waltham, MA, USA). The analyses were performed in KED (Kinetic Energy Discrimination) mode using Helium as collision gas. The solutions were prepared using deionized water, HNO_3_ (65% m/m), and HCl (37% m/m). The concentrations of the analytes were estimated using calibration lines (CertiPUR^®^, Merck, Darmstadt, Germany) (r^2^ > 0.98). For the evaluation of Polycyclic aromatic hydrocarbons (PAHs), the extraction was performed with solid-phase extraction (SPE) by Oasis HLB cartridge (6 mL, 500 mg; Waters, Milford, USA), according to the method proposed by Zhou et al. [65]. Before extraction, benzo[a]pyrene-d_12_, and indeno [1,2,3-cd]pyrene-d_12_ were added as surrogate solutions. Methylene chloride (5 mL), methanol (5 mL), and ultra-pure water (5 mL) were used to condition and wash the cartridge, and then the water sample was eluted with a flow rate of 10 mL min^−1^. Therefore, the cartridge was eluted with methylene chloride (10 mL), and the extract was concentrated to 500 µL in hexane for GC-MS analysis. Finally, chrysene-d_12_ was added to the sample as an internal standard. In order to perform the Organochlorine and Organophosphorus Pesticides (OCLs and OPPs) and the Polychlorinated biphenyls (PCBs) extraction, water samples were adjusted to pH~7 and preconcentrated using SPE Oasis HLB cartridges (6 mL, 500 mg; Waters, Milford, USA), previously preconditioned with 5 mL of ethyl acetate (EtOAc), 5 mL of methanol (MetOH) and 2.5 mL of deionized water [66]. The extracts were eluted with 6 mL of EtOAc, evaporated to dryness, and reconstituted in hexane for analysis by GC-MS. Semi-volatiles organic compounds (SVOCs) surrogate standard (2-fluorobiphenyl, nitrobenzene-d_5_, *p*-terphenyl-d_14_, 2-fluorophenol, phenol-d_5_, 2,4,6-tribromophenol) and SVOCs internal standard (acenaphtene-d_10_, crysene-d_12_, 1,4-dichlorobenzene-d_4_, naphtalene-d_8_, perylene-d_12_, phenantrene-d_10_) mixtures were used as surrogate and internal standards, respectively. For determining trihalomethanes (THMs) in water samples, purge-and-trap (PT) extraction followed by gas chromatography–mass spectrometric (GC–MS) analysis were used. For analysis of PAHs, OCLs, OPPs, PCBs, and THMs, a TRACE^TM^ 1310 Gas Chromatography coupled to ISQTM 7000 Single Quadrupole Mass Spectrometer (GC-MS, Thermo Scientific, USA) under selected-ion monitoring (SIM) mode was used to perform the analysis. A TG-5MS capillary column (30 mm length × 0.25 mm inner diameter × 0.25 μm film thickness) and helium as carrier gas (constant flow rate of 1 mL/min) were used. The MSD worked in the electron ionization (EI) mode, set at 70 eV. Splitless injection mode was adopted, and sample injection volume was 1 μL. To check the methods, all samples were analyzed in duplicate. Quality assurance and quality control were assessed using duplicates, method blanks, and standard reference materials. The accuracy of the instrumental methods was checked by triplication of the samples, as well as by using spiked samples, which was run after every single sample. The limit of detections (LODs) and limit of quantifications (LOQs) were estimated as 3 and 10 times, respectively, the signal/noise ratio for each analyte by five replicate analyses. The recoveries of detected analytes in spiked samples were in the range of 70–130%, which met the quality control requirements.

### 4.3. Cell Cultures

BJ cells (human healthy fibroblasts established from skin taken from normal foreskin of a neonatal male, ATCC CRL-2522) and HaCaT cells (normal human keratinocytes, ATCC CRL-2404) were maintained in Dulbecco’s modified essential medium (DMEM, Gibco, Thermo Fisher Scientific, Inc., Waltham, MA, USA) with L-glutamine, supplemented with 10% FCS (fetal calf serum, Gibco), and 1% antibiotic (Penicillin and Streptomicin, Gibco). All cultured cells were kept at 37 °C in a humidified atmosphere of 95% air and 5% of carbon dioxide (CO_2_). When the cells reached confluence, the culture medium was removed from the flask and cells were rinsed two times with sterile PBS (Phosphate-Buffered Saline, Gibco). The confluent layer was trypsinized using Trypsin/EDTA (Gibco) and then resuspended in fresh medium.

### 4.4. Preparation of Nitrodi’ Water Solution

To analyze the biological effects of Nitrodi’s water in the cultured cells, the following salts that make up PBS were dissolved in 1.0 L of Nitrodi’s water: 80 g of NaCl; 2.0 g of KCl; 14.4 g of Na_2_HPO_4_; and 2.4 g of KH_2_PO_4_. After mixing to dissolve, the pH was adjusted to 7.2. This solution, indicated in the text and in the figures as Nitrodi, was diluted 1:10 and stored at room temperature. Cells treated with PBS solution (prepared in distilled water) were used as negative control. Both Nitrodi’s water and PBS solution were sterilized by filtration using 0.22 μm membrane filters.

### 4.5. Western Blot Analysis

Immunoblotting experiments were performed according to standard procedures [67]. Briefly, cells were harvested in lysis RIPA buffer (RIPA Buffer: 20 mM Tris-HCl (pH 7.5), 150 mM NaCl, 1 mM Na_2_ EDTA, 1 mM EGTA, 1% NP-40, 1% sodium deoxycholate, 2.5 mM sodium pyrophosphate, 1 mM β-glycerophosphate, 1 mM Na_3_VO_4_, and 1 µg/mL leupeptin) supplemented with a mixture of proteases and phosphatases inhibitors. Thirty micrograms of protein were electrophoresed on a 10% SDS-PAGE and transferred onto a polyvinylidene fluoride membrane. The membrane was blocked with 5% nonfat dry milk and probed with specific Abs: mouse anti-p-ERK 1/2 (1 μg/mL), rabbit anti-ERK (1 μg/mL), mouse anti-alpha-SMA (2 μg/mL), and rabbit anti-β-actin (0.5 μg/mL). Finally, washed filters were incubated with HRP-conjugated anti-rabbit or anti-mouse antibodies. The immunoreactive bands were detected by a chemiluminescence kit and quantified by densitometry (ChemiDoc XRS, Bio-Rad). Quantifications of Western blot were performed using ImageJ software version 1.53 m (National Institute of Health, Bethesda, MD, USA).

### 4.6. Proliferation Assay

Cultured cells and HaCaT cells were serum-starved overnight using DMEM 0.1% BSA, plated at 5 × 10^3^ cells/well in 96-well plates (Corning), and then treated with Nitrodi supplemented with 0.5% BSA (starving condition) and 10% FCS (growing condition). Cells treated with PBS supplemented with 0.5% BSA and 10% FCS were used as a negative control. Cells treated with culture medium supplemented with 0.5% BSA and 10% FCS were used as a positive control. In experiments on the role of ERKs in BJ cell proliferation, cultured cells were pre-treated with the ERK 1/2 inhibitor PD98059 at 50 μM for 1 h at 37 °C and then exposed to PBS, Nitrodi, or culture medium.

The cell proliferation was measured at different time points, as showed in the figures. At the end of the incubation, 20 μL/well CellTiter-96 was added. After incubation at 37 °C for 2 h, the absorbance was determined by an ELISA reader (Bio-Rad) at a wavelength of 490 nm according to the manufacturer’s instructions.

### 4.7. Chemotaxis Assay

Chemotaxis assays were performed using a modified Boyden chamber technique [68]. Briefly, 25 μL of medium supplemented with 5% FCS were placed in triplicate in the lower compartment of a microchemotaxis chamber (NeuroProbe, Cabin John, MD, USA). The lower compartments were covered with 8-μm-pore polycarbonate membranes coated with fibronectin (10 μg/mL). Fifty microliters of the cell suspension (5 × 10^4^/well), resuspended in PBS (as a negative control) and Nitrodi, were loaded into the upper compartments. The chemotactic chamber was then incubated for 24 h at 37 °C in a humidified incubator with 5% CO_2_. Then, the membrane was removed, the upper side was washed with PBS, and cells attached to the lower surface of the filter were fixed, stained with May-Grünwald-Giemsa, mounted on a microscope slide with Cytoseal (Stephens Scientific, Springfield, NJ, USA), and counted. In each experiment, 10 fields/triplicate filters were measured at ×40 magnification.

### 4.8. Wound Healing Assay

BJ cells and HaCaT cells were seeded into a 12-well culture plate using DMEM containing 10% FCS and incubated for 12 h at 37 °C to create a confluent monolayer. Cells were then scraped with a p200 pipette tip in a straight line to create a “scratch.” The debris was removed, and the edge of the scratch was smoothed by washing cells once with 2 mL growth medium. Cells were then incubated at 37 °C with PBS (as a negative control) or Nitrodi for different time points, at baseline (T = 0), 24, and 72 h. The data acquisition was conducted through microscopic-image capturing and gap measurement at each time point. Measurements of wound length were carried out manually using ImageJ software (National Institutes of Health, Bethesda, MD, USA). Three measurements of length for each digital picture were performed in order to make an accurate measurement of the image.

### 4.9. In Situ Trypan Blue Staining

To assess the viability of HaCaT cells subjected to wound healing assay (as described above), the cells were incubated with 0.4% trypan blue solution. The cells were washed with PBS for three times, and then incubated with trypan blue for 10 min at room temperature. After trypan blue staining, cells were fixed with 4% paraformaldehyde [69,70]. Stained and fixed cells were observed by 10× objective of inverted-phase contrast microscope (Olympus CKX41).

### 4.10. In Situ ELISA

A quantitative assessment of extracellular matrix (ECM) proteins (vitronectin, fibronectin, and collagen type I) was performed by in situ ELISA. BJ cells were plated in 96-well plates (Corning) at a density of 5 × 10^4^ cells per well and treated with PBS (as a negative control) and Nitrodi. In parallel, the plates were coated with purified ECM proteins (vitronectin, fibronectin, and collagen type I) at different concentrations (16, 8, 4, 2, 1, and 0 μg/mL), in order to provide a quantification of absorbed ECM proteins on the tissue culture plastic. The concentration of the test samples was determined by using their absorbance values and interpolating this from the calibration curve. After 24 h of incubation, proteins were fixed using acetone/methanol (*v*/*v*) for 10 min at 22 °C, incubated in 0.5% PBS/BSA and 0.2% Tween 20 for 30 min at 22 °C to minimize aspecific binding sites, and washed in PBS. Goat polyclonal anti-vitronectin (2 μg/mL) antibody, mouse monoclonal anti-fibronectin (2 μg/mL), and anti-collagen type 1 (2 μg/mL) antibodies were added for 1 h at 22 °C. After three washes in PBS, plates were incubated with HRP secondary antibodies for 30 min at 22 °C. After three washes in PBS, the substrate was added (1 mg/mL OFD, 0.1 mol/l citrate buffer [pH 5], and 0.006% H_2_O_2_), and plates were incubated for 30 min at 37 °C in the dark. The reaction was then stopped by 1 M H_2_SO_4_, and the absorbance was read at 450 nm by a spectrophotometer.

### 4.11. Reactive Oxygen Species Detection

Cells were plated overnight at 2 × 10^4^ cells/well in 96-well plates using DMEM with 10% FCS. Cells were incubated with DCFH-DA at a concentration of 5 µM for 30 min in the dark at 37 °C. The esterified form of DCFH-DA can permeate cell membranes before being deacetylated by intracellular esterases. The resulting compound, dichlorodihydrofluorescein, reacts with reactive oxygen species (ROS), producing an oxidized fluorescent compound, dichlorofluorescein (DCF), which can be detected using a multiplate reader. After incubation with DCFH-DA, cells were washed twice and treated with H_2_O_2_ (1 mM) or fMLF (10^−4^ M) in presence of PBS (as a negative control) and Nitrodi for 5, 15, 30, and 60 min at 37 °C in a humidified 5% CO_2_ incubator. DCF was detected at a wavelength of 535 nm by a microplate reader (Tecan Trading AG, Switzerland). DCFH-DA-unloaded cells were examined in parallel and subtracted to DCFH-DA-loaded cell values.

### 4.12. Statistical Analysis

All the experiments were performed at least in triplicate. The results were expressed as mean ± SEM. Values from groups were compared using a paired Student *t*-test [71]. Differences were considered significant when * *p* < 0.05 and ** *p* < 0.001.

## Figures and Tables

**Table 1:** Mineral composition of Nitrodi’s water.

Parameters	Unit	Results
Water temperature at source	°C	+28.4
pH at source	/	6.33
Silica (SiO_2_)	mg/L	82
Bicarbonate (HCO_3_^−^)	mg/L	561
Chlorides (Cl^−^)	mg/L	93
Sulphates (SO_4_^2−^)	mg/L	204
Sodium (Na^+^)	mg/L	174
Potassium (K^+^)	mg/L	21
Calcium (Ca^2+^)	mg/L	137
Magnesium (Mg^2+^)	mg/L	17
Iron (dissolved) (Fe^2+^, Fe^3+^)	mg/L	<0.02
Ammonium (NH_4_^+^)	mg/L	<0.02
Phosphorus (P total)	mg/L	<0.05
Stronzium (Sr^2+^)	mg/L	0.31
Lithium (Li^+^)	mg/L	0.04
Aluminium (Al^3+^)	mg/L	<0.02
Bromide (Br^−^)	mg/L	0.18
Iodide (I^−^)	mg/L	<0.02
PAHs	μg/L	0.025
OCLs	μg/L	0.01
OPPs	μg/L	0.01
PCBs	μg/L	0.0025
THMs	μg/L	0.1

## Data Availability

The data underlying this article will be shared upon reasonable request to the corresponding author.
